# The association between fear of COVID-19 and depression, anxiety, and psychosis among Lebanese chronic patients with schizophrenia: any moderating effect of spirituality?

**DOI:** 10.3325/cmj.2022.63.412

**Published:** 2022-10

**Authors:** Zeinab Bitar, Chadia Haddad, Sahar Obeid, Souheil Hallit

**Affiliations:** 1 Faculty of Sciences, Lebanese University, Fanar, Lebanon; 2 Research Department, Psychiatric Hospital of the Cross, Jal Eddib, Lebanon; 3 Institut National de Santé Publique, d’Épidémiologie Clinique et de Toxicologie-Liban, Beirut, Lebanon; 4 School of Health Sciences, Modern University for Business and Science, Beirut, Lebanon; 5 Social and Education Sciences Department, School of Arts and Sciences, Lebanese American University, Jbeil, Lebanon; 6 School of Medicine and Medical Sciences, Holy Spirit University of Kaslik, Jounieh, Lebanon

## Abstract

**Aim:**

To assess whether fear of coronavirus disease 2019 (COVID-19) is associated with depression, anxiety, and psychosis and to evaluate if these variables are correlated with the interaction between spirituality and fear of COVID-19.

**Methods:**

Between September and November 2020, this cross-sectional study enrolled 118 chronic schizophrenia patients. The interview with patients included Fear of COVID-19 Scale, Lebanese Anxiety Scale-10, Montgomery-Asberg Depression Rating Scale, Positive and Negative Syndrome Scale (PANSS), and Functional Assessment of Chronic Illness Therapy-Spiritual Well-Being-12. The results were analyzed by using linear regressions (Enter method), with anxiety, depression, total PANSS score, positive PANSS, negative PANSS, and general psychopathology PANSS subscales as dependent variables. Spirituality, fear of COVID-19, and the interaction of spirituality with fear of COVID-19 were independents variables.

**Results:**

Fear of COVID-19 was positively correlated with increased total PANSS scores (Beta = 0.90, *P* = 0.030). Higher spirituality was significantly associated with lower anxiety (Beta = -0.14, *P* = 0.009), lower depression (Beta = -0.21, *P* = 0.001), lower total PANSS score (Beta = -0.90, *P* = 0.004), lower negative PANSS score (Beta = -0.23, *P* = 0.009), and lower general psychopathology PANSS score (Beta = -0.61, *P* = 0.001). In patients with high fear of COVID-19, having low spirituality was significantly associated with higher anxiety, depression, and psychotic symptoms.

**Conclusion:**

This study suggests a positive correlation between fear of COVID-19 and higher psychosis among inpatients with schizophrenia. The interaction of spirituality with fear of COVID-19 was correlated with reduced anxiety, depression, and psychosis.

The impact of the SARS-CoV-2 on the central nervous system and the associated neuropsychiatric consequences have not been widely studied. Various types of neuropsychiatric symptoms can emerge during or after an acute infection with a respiratory virus (1-5).

An effect of having a psychiatric condition on the vulnerability to coronavirus disease 2019 (COVID-19) has also been reported. Patients who already had a psychiatric disorder had 65% higher incidence of COVID-19 (6). This association was explained by behavioral factors arising from psychiatric conditions such as less adherence to restrictions or by socioeconomic and lifestyle variables such as smoking (6). Additionally, COVID-19 vulnerability might be aggravated by the pro-inflammatory state associated with psychotropic medications use or certain psychiatric disorders (6).

Fear of COVID-19 increases the incidence of anxiety and depression in the general population (7), as well as in individuals previously diagnosed with psychiatric disorders (8-10). Fear of COVID-19 was also correlated with female gender and the presence of chronic diseases (8). It was a risk factor for the development of psychotic symptoms correlated with a higher probability of psychotic relapse and a lower stable diagnosis (11).

In 1995, spirituality was added as a psychosocial environmental factor in the Diagnostic and Statistical Manual of Mental Disorders (DSM)-IV (12). Spirituality relieves the sense of dissatisfaction and emptiness in one’s life (13), but may have some disadvantages such as loss of compassion and social solidarity (14). In general, spiritual patients with schizophrenia have shown increased social integration, better quality of life and recovery, reduced risk of suicide, and decreased substance abuse and smoking (15). During stressful situations, individuals frequently turn to spirituality for support and comfort (16). Spirituality has been linked to measures of well-being and has been shown to ameliorate sadness and anxiety in individuals with schizophrenia (17,18). Individuals with schizophrenia may use spiritual coping to facilitate problem-solving during times of stress (19); spiritual coping consists of spiritual beliefs or behaviors that prevent the negative mental consequences of stressful life situations (15). On the other hand, negative spiritual coping increases depression, anxiety, and distress among psychiatric patients (20). The association between spirituality and psychosis has not been investigated in depth (21). However, spirituality appears to be useful for patients with psychosis (22). During the COVID-19 pandemic, increased spirituality was correlated with lower psychosis (23), lower anxiety, and depression (24,25). However, to the best of our knowledge, few studies evaluated the interaction between fear of COVID-19 and spirituality and its association with depression, anxiety, and psychosis among psychiatric patients. During the COVID-19 pandemic, Lebanese people were faced with many multifactorial challenges to the mental health (26). Therefore, the aim of this research was to evaluate the moderating effect of spirituality in the association between fear of COVID-19 and depression, anxiety, and psychosis.

## METHODS

### Study design

**Figure 1:**
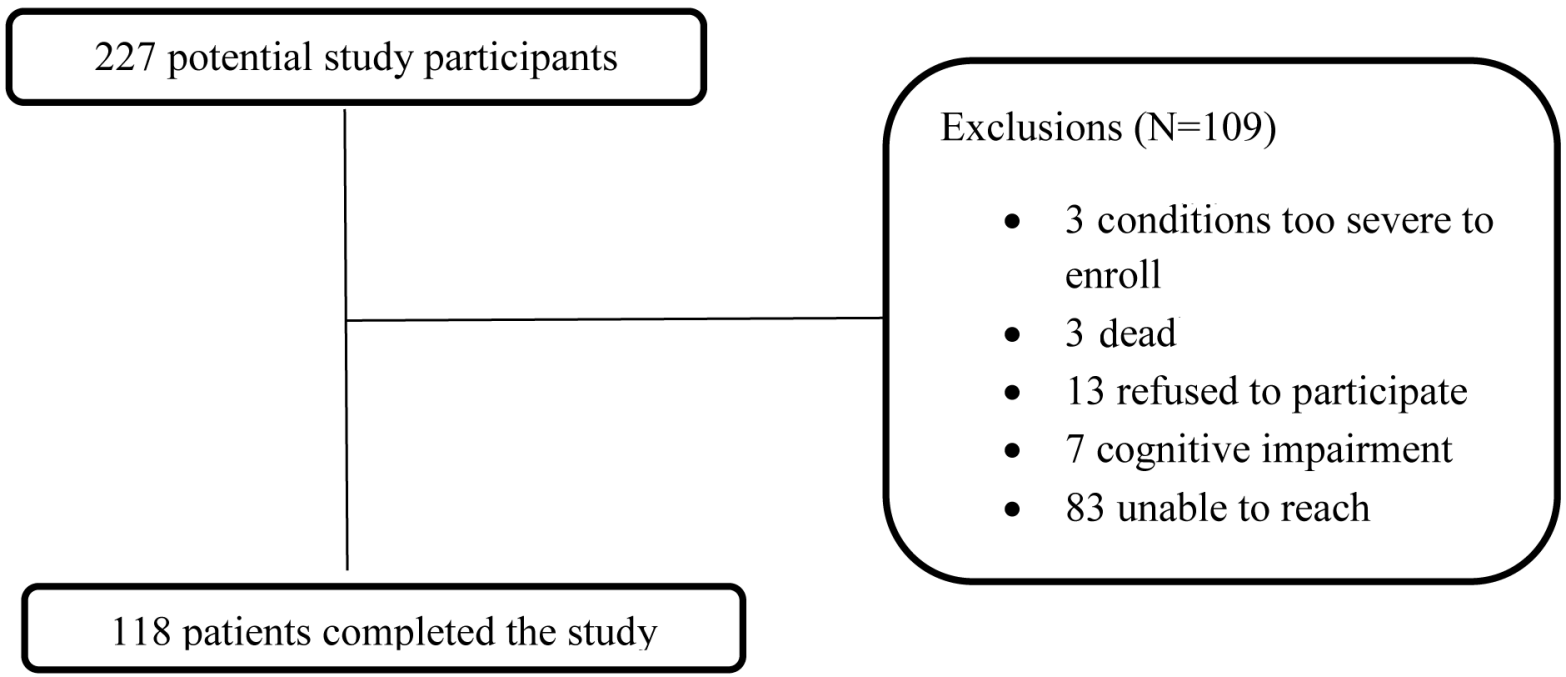
Study flowchart.

This cross-sectional study was performed between September and November 2020. The inclusion criteria were chronic schizophrenia, age above 18 years, and hospital stay for more than one year in Psychiatric Hospital of the Cross, Jal el Dib, Lebanon. Schizophrenia was diagnosed by a clinician according to the DSM-5. The exclusion criteria were clinical conditions preventing the patients from participating in the interviews (at the physician's discretion), schizoaffective disorder (based on the DSM-5 criteria), cognitive impairment (according to the Mini-Mental State Exam), delusional disorder (patients unable to answer correctly), and refusal to answer the questions (27). The final sample consisted of 118 respondents (Figure 1).

The study was approved by Psychiatric Hospital of the Cross Ethics and Research Committee and it complied with the Hospital's Regulatory Research Protocol. A written informed consent was obtained from each patient.

### Data collection and measurement

The questionnaire was in Arabic, with the response time from 30 to 45 minutes. The data were collected by a trained person through a personal interview. The first section of the questionnaire inquired about socio-demographic variables. The second section included questions inquired about medical history and medications used.

Fear of COVID-19 was assessed with the seven-item Fear of COVID-19 Scale (FCV-19S). In this scale, the questions are graded from one to five, with higher scores indicating more fear of COVID-19 (28). The Cronbach’s alpha in this study was 0.91.

Anxiety level was assessed with the 10-item Lebanese Anxiety Scale-10 (LAS-10). It is a scale measuring the severity of anxiety symptoms among Lebanese adults (29) and adolescents (30). Questions 1 to 7 were graded from one to ten, and questions 8 to 10 were graded from one to four based on the repetitive manifestation of symptoms. The Cronbach's alpha in this study was 0.81.

Depression level was assessed with the Montgomery-Asberg Depression Rating Scale (MADRS). The 10-item scale, with each item presenting a group of symptoms (31), is an instrument used to assess the total level of depression, shown to be reliable among Lebanese psychiatric patients (32). The scale evaluates mood symptoms during the previous two weeks. Questions in this instrument are graded from zero to six with a maximum of 60 points. The higher the MADRS scores, the more severe the depressive symptoms (32). The Cronbach’s alpha in this study was 0.78.

Spiritual well-being was assessed with the self-rated, 12-item Functional Assessment of Chronic Illness Therapy-Spiritual Well-Being-12 (FACIT-SP12). All items in this scale are graded from zero to four according to their occurrence during the previous seven days. The responses are summed up to create a total FACIT-SP-12 score. Higher scores reflect higher spiritual well-being (33). The Cronbach’s alpha in this study was 0.97.

Positive and negative symptoms of psychotic disorder were evaluated with the 30-item Positive and Negative Syndrome Scale (PANSS) (34). The Arabic version of PANSS has been proved reliable and valid in Lebanon (35). PANSS items are graded on a seven-point Likert scale. The total score is obtained by summing up the answers to each question. A higher score shows a higher level of symptom severity (34). The Cronbach’s alpha values in this study were as follows: total scale (0.97), positive PANSS subscale (0.85), negative PANSS subscale (0.88), and general psychopathology subscale (0.96).

### Statistical analysis

Using the G-power version 3.1.9.4 software, we calculated a minimum required sample of 82 participants, according to a hypothetical correlation effect size (since no similar international studies are available) of 0.3, an alpha error of 5%, and a power of 80%.

Cronbach’s alpha values were calculated for all the scales and subscales. Missing data were not replaced as they represented less than 5% of the database. The normality of distribution of depression, anxiety, and PANSS total scale and subscale scores was confirmed by visual inspection of the histogram and calculation of the skewness and kurtosis. Values for skewness and kurtosis between -2 and +2 indicated normal univariate distribution (36). The normality of distribution was additionally checked by assessing the line of the regression plot and the scatter plot of the residual.

Differences between two or three groups were assessed with a *t* test or ANOVA, respectively. The correlation was assessed with a Pearson correlation test. Six linear regressions were conducted by using the Enter method with the following dependent variables: anxiety, depression, total PANSS score, positive PANSS, negative PANSS, general psychopathology PANSS subscales. Two models were used. In the first model, the independent variables were spirituality and fear of COVID-19 scales. In the second model (multiplicative interaction analysis), the interaction between spirituality and fear of COVID-19 was added. The variables showing a significant *P* value in the bivariate analysis were entered as adjusted variables. *P* < 0.05 was considered significant. Statistical data analysis was performed with the SPSS version 25 (IBM Corp., Armonk, NY, USA).

## RESULTS

The average age was 51.78 ± 14.61 years (Table 1). There were 50.8% of women; the majority of respondents were single (91.5%) and had no income (72.0%).

**Table 1:** Respondents' (N = 118) socio-demographic characteristics

	N	%
**Sex**		
female	60	50.8
male	58	49.2
**Education level**		
none	16	14.0
elementary	17	14.9
intermediate	31	27.2
secondary	29	25.4
university	21	18.4
**Marital status**		
single	108	91.5
married	10	8.5
**Monthly income**		
no	85	72.0
yes	33	28.0
**Place of living**		
urban	92	82.1
rural	20	17.9
**Age (in years); mean and standard deviation**	51.78	14.61

Married participants had higher anxiety compared with single patients (19.10 vs 13.14, *P* < 0.001). Respondents with intermediate education level had higher anxiety compared with respondents with other education levels. Fear of COVID-19 was significantly positively correlated with anxiety (r = 0.20, *P* = 0.033), whereas spirituality was inversely correlated with anxiety (r = -0.48, *P* < 0.001).

Married patients had significantly higher depression compared with single respondents (13.60 vs 8.67, *P* = 0.025) and respondents living in rural areas had higher depression than those living in urban areas (12.70 vs 7.55, *P* < 0.001). Spirituality was significantly inversely correlated with depression (r = -0.53, *P* < 0.001). Married respondents compared with single respondents, respondents living in rural areas compared with those living in urban areas, and respondents who had an elementary education level compared with respondents with other educational levels had significantly higher mean total PANSS, negative PANSS, and general psychopathology PANSS scores. Spirituality was significantly inversely correlated with total, positive, negative, and general psychopathology PANSS scores. However, fear of COVID-19 was significantly positively correlated with total PANSS scale and subscales scores.

**Figure 2:**
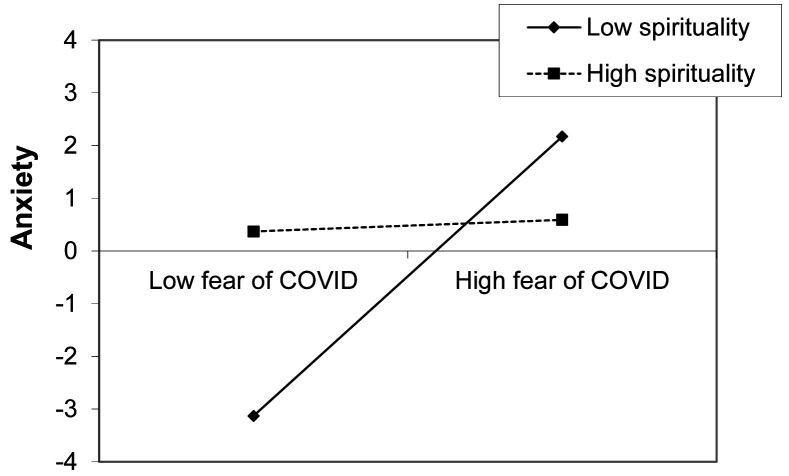
Association between anxiety and the interaction of fear of COVID-19 with spirituality.

In the multiple regression, less anxiety was significantly associated with more spirituality (Beta = -0.14, *P* = 0.014); however anxiety was not associated with fear of COVID-19 (Table 2). The model to which interaction between fear of COVID-19 and spirituality was added explained the variation of anxiety better (R^2^ = 42%) than the model 1 (R^2^ = 39%); in patients with high fear of COVID-19, having low spirituality was significantly associated with higher anxiety (Figure 2).

**Table 2:** Linear regression analysis with the anxiety scale as the dependent variable

		Unstandardized Beta	Standardized Beta	p	Confidence interval	
					lower bound	upper bound
Model 1	Fear of COVID-19 scale	0.04	0.04	0.619	-0.11	0.18
	Age	0.06	0.17	**0.041**	0.00	0.12
	Gender (male* vs female)	-4.21	-0.39	**<0.001**	-6.25	-2.16
	Place of living	1.06	0.07	0.403	-1.45	3.58
	Marital status (married vs single*)	4.17	0.22	**0.009**	1.08	7.25
	Education (complementary vs primary*)	1.42	0.12	0.222	-0.87	3.71
	Education (secondary vs primary*)	-0.46	-0.04	0.699	-2.81	1.90
	Education (university vs primary*)	-2.08	-0.15	0.117	-4.68	0.53
	Spirituality index scale	-0.14	-0.24	**0.009**	-0.25	-0.04
Adjusted R^2^ = 0.39						
Model 2	Fear of COVID-19 scale	0.81	0.99	**0.014**	0.17	1.45
	Age	0.06	0.17	**0.036**	0.00	0.12
	Gender (male* vs female)	-3.92	-0.36	**<0.001**	-5.92	-1.91
	Place of living	0.90	0.06	0.470	-1.56	3.35
	Marital status (married vs single*)	3.38	0.17	**0.032**	0.30	6.45
	Education (complementary vs primary*)	1.26	0.10	0.264	-0.97	3.50
	Education (secondary vs primary*)	-0.25	-0.02	0.833	-2.55	2.06
	Education (university vs primary*)	-1.86	-0.14	0.149	-4.41	0.68
	Spirituality index scale	0.21	0.34	0.177	-0.10	0.51
	Interaction Spirituality by Fear of Covid-19 scale	-0.02	-1.00	**0.016**	-0.04	-0.00
Adjusted R^2^ = 0.42						
Adjusted variables: age, gender, place of living, education level and marital status.						

*reference category.

**Figure 3:**
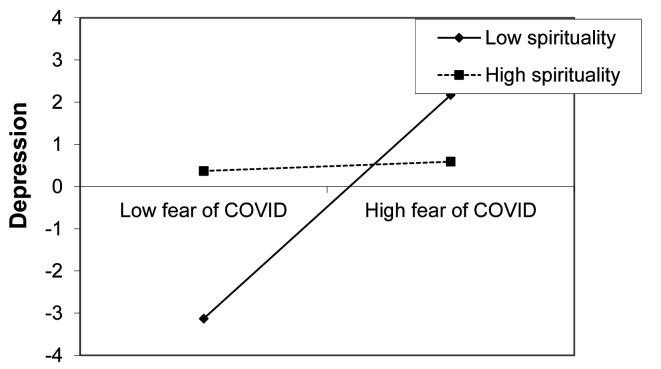
Association between depression and the interaction of fear of COVID-19 with spirituality.

Lower depression was significantly associated with more spirituality (Beta = -0.21, *P* < 0.001); however, depression was not associated with fear of COVID-19 (Table 3). The model to which the interaction between fear of COVID-19 and spirituality was added explained the variation in depression better (R^2^ = 33.0%) than model 1 (R^2^ = 30%); in patients with high fear of COVID-19, having low spirituality was significantly associated with higher depression (Figure 3).

**Table 3:** Linear regression analysis with the depression scale as the dependent variable

		Unstandardized Beta	Standardized Beta	p	Confidence interval	
					lower bound	upper bound
Model 1	Fear of COVID-19 scale	-0.02	-0.02	0.821	-0.18	0.14
	Age	0.05	0.12	0.154	-0.02	0.12
	Gender (male* vs female)	-2.68	-0.23	**0.025**	-5.01	-0.35
	Place of living	-2.27	-0.14	0.119	-5.13	0.60
	Marital status (married vs single*)	3.54	0.17	**0.048**	0 .03	7.06
	Education (complementary vs primary*)	-0.25	-0.02	0.848	-2.86	2.36
	Education (secondary vs primary*)	-0.15	-0.01	0.914	-2.83	2.54
	Education (university vs primary*)	-1.74	-0.12	0.248	-4.70	1.23
	Spirituality index scale	-0.21	-0.33	**0.001**	-0.34	-0.09
Adjusted R^2^ = 0.30						
Model 2	Fear of COVID-19 scale	0.78	0.89	**0.038**	0.05	1.52
	Age	0.05	0.12	0.148	-0.02	0.11
	Gender (male* vs female)	-2.38	-0.20	**0.043**	-4.68	-0.08
	Place of living	-2.44	-0.16	0.088	-5.25	0.37
	Marital status (married vs single*)	2.73	0.13	0.127	-0.79	6.25
	Education (complementary vs primary*)	-0.41	-0.03	0.751	-2.97	2.15
	Education (secondary vs primary*)	0.07	0.01	0.956	-2.56	2.71
	Education (university vs primary*)	-1.52	-0.11	0.304	-4.43	1.40
	Spirituality index scale	0.15	0.23	0.403	-0.20	0.49
	Interaction Spirituality by Fear of Covid-19 scale	-0.02	-0.97	**0.030**	-0.04	-0.00
Adjusted R^2^ = 0.33						
Adjusted variables: Age, gender, place of living, education level, and marital status.						

*reference category.

**Figure 4:**
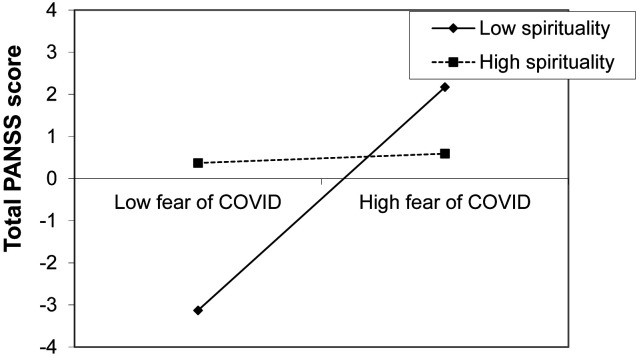
Association between Positive and Negative Syndrome Scale (PANSS) scores and the interaction of fear of COVID-19 and spirituality.

Lower total PANSS score was associated with more spirituality (Beta = -0.90, *P* < 0.001) (Table 4). Increased PANSS score was associated with higher fear of COVID-19 (Beta = 0.9, *P* = 0.03). The model to which the interaction between fear of COVID-19 and spirituality was added explained the variation in the total PANSS score better (R^2^ = 49%) than model 1 (R^2^ = 44%); in patients with high fear of COVID-19, having low spirituality was significantly associated with a higher total PANSS score (Figure 4).

**Table 4:** Linear regression analysis with the total Positive and Negative Syndrome Scale as the dependent variable

		Unstandardized Beta	Standardized Beta	p	Confidence interval	
					lower bound	upper bound
Model 1	Fear of COVID-19 scale	0.90	0.18	**0.030**	0.09	1.71
	Age	-0.02	-0.01	0.898	-0.36	0.32
	Gender (male* vs female)	-19.29	-0.27	**0.001**	-30.97	-7.61
	Place of living	-9.48	-0.11	0.193	-23.84	4.88
	Marital status (married vs single*)	18.09	0.16	**0.044**	0.48	35.71
	Education (complementary vs primary*)	1.86	0.03	0.779	-11.22	14.94
	Education (secondary vs primary*)	-14.80	-0.20	**0.031**	-28.25	-1.35
	Education (university vs primary*)	-3.46	-0.04	0.646	-18.33	11.42
	Spirituality index scale	-0.91	-0.25	**0.004**	-1.52	-0.30
Adjusted R^2^ = 0.44						
Model 2	Fear of COVID-19 scale	6.74	1.38	**<0.001**	3.16	10.32
	Age	-0.02	-0.01	0.885	-0.34	0.30
	Gender (male* vs female)	-17.08	-0.26	**0.003**	-28.27	-5.89
	Place of living	-10.76	-0.12	0.122	-24.43	2.92
	Marital status (married vs single*)	12.14	0.11	0.163	-5.00	29.27
	Education (complementary vs primary*)	0.70	0.01	0.912	-11.77	13.16
	Education (secondary vs primary*)	-13.18	-0.18	**0.044**	-26.01	-0.35
	Education (university vs primary*)	-1.86	-0.02	0.796	-16.04	12.33
	Spirituality index scale	1.73	0.48	**0.044**	0.05	3.41
	Interaction Spirituality by Fear of Covid-19 scale	-0.15	-1.27	**0.001**	-0.25	-0.06
Adjusted R^2^ = 0.49						
Adjusted variables: Age, gender, place of living, education level and marital status.						

*reference category.

No significant association was found between positive PANSS subscale, spirituality, and fear of COVID-19 (Table 5). The model to which the interaction between fear of COVID-19 and spirituality was added explained additional 5.1% of the variation in the positive PANSS score; the interaction between spirituality and fear of COVID-19 was significantly associated with lower positive PANSS score (Beta = -0.04, *P* = 0.010).

**Table 5:** Linear regression analysis with the positive Positive and Negative Syndrome Scale score as the dependent variable

		Unstandardized Beta	Standardized Beta	p	Confidence interval	
					lower bound	upper bound
Model 1	Fear of COVID-19 scale	0.13	0.11	0.304	-0.12	0.37
	Age	-0.04	-0.07	0.466	-0.14	0.06
	Gender (male* vs female)	-1.77	-0.11	0.313	-5.23	1.70
	Place of living	-1.48	-0.07	0.493	-5.73	2.78
	Marital status (married vs single*)	4.43	0.16	0.096	-0.78	9.65
	Education (complementary vs primary*)	1.65	0.10	0.401	-2.23	5.53
	Education (secondary vs primary*)	-3.44	-0.19	0.090	-7.43	0.55
	Education (university vs primary*)	1.04	0.05	0.641	-3.37	5.45
	Spirituality index scale	-0.06	-0.07	0.484	-0.25	0.12
Adjusted R^2^ = 0.13						
Model 2	Fear of COVID-19 scale	1.52	1.31	**0.006**	0.44	2.61
	Age	-0.03	-0.07	0.448	-0.14	0.06
	Gender (Male* vs Female)	-1.24	-0.08	0.469	-4.63	2.15
	Place of living	-1.78	-0.09	0.395	-5.92	2.36
	Marital status (married vs single*)	3.00	0.1	0.253	-2.18	8.18
	Education (complementary vs primary*)	1.37	0.08	0.473	-2.40	5.14
	Education (secondary vs primary*)	-3.06	-0.17	0.121	-6.94	0.83
	Education (university vs primary*)	1.42	0.07	0.512	-2.87	5.71
	Spirituality index scale	0.57	0.66	**0.029**	0.06	1.08
	Interaction Spirituality by Fear of Covid-19 scale	-0.04	-1.28	**0.010**	-0.07	-0.01
Adjusted R^2^ = 0.18						
Adjusted variables: Age, gender, place of living, education level and marital status.						

*reference category.

Lower negative PANSS score was significantly associated with higher spirituality (Beta = -0.23, *P* = 0.01); however negative PANSS was not significantly associated with fear of COVID-19 (Table 6). The model to which the interaction between fear of COVID-19 and spirituality was added explained the variation in the negative PANSS score better (R^2^ = 46%) than model 1 (R^2^ = 43%); the interaction between spirituality and fear of COVID-19 was significantly associated with lower negative PANSS score (Beta = -0.03, *P* = 0.02).

**Table 6:** Linear regression analysis with the negative Positive and Negative Syndrome Scale score as the dependent variable

		Unstandardized Beta	Standardized Beta	p	Confidence interval	
					lower bound	upper bound
Model 1	Fear of COVID-19 scale	0.12	0.09	0.313	-0.11	0.35
	Age	-0.02	-0.03	0.663	-0.12	0.07
	Gender (Male* vs Female)	-7.30	-0.40	**<0.001**	-10.59	-4.01
	Place of living	-3.34	-0.14	0.104	-7.39	0.70
	Marital status (married vs single*)	4.02	0.12	0.111	-0.94	8.98
	Education (complementary vs primary*)	-1.85	-0.10	0.322	-5.53	1.84
	Education (secondary vs primary*)	-3.79	-0.18	0.050	-7.58	0.00
	Education (university vs primary*)	0.26	0.01	0.902	-3.93	4.45
	Spirituality index scale	-0.23	-0.23	**0.010**	-0.40	-0.06
Adjusted R^2^ = 0.43						
Model 2	Fear of COVID-19 scale	1.31	0.96	**0.014**	0.28	2.35
	Age	-0.02	-0.03	0.651	-0.11	0.07
	Gender (Male* vs Female)	-6.85	-0.38	**<0.001**	-10.09	-3.61
	Place of living	-3.60	-0.15	0.074	-7.56	0.35
	Marital status (married vs single*)	2.80	0.09	0.264	-2.15	7.76
	Education (complementary vs primary*)	-2.09	-0.10	0.254	-5.69	1.52
	Education (secondary vs primary*)	-3.46	-0.16	0.068	-7.17	0.26
	Education (university vs primary*)	0.59	0.03	0.776	-3.51	4.69
	Spirituality index scale	0.31	0.31	0.208	-0.18	0.80
	Interaction Spirituality by Fear of Covid-19 scale	-0.03	-0.93	**0.021**	-0.06	-0.01
Adjusted R^2^ = 0.46						
Adjusted variables: Age, gender, place of living, education level and marital status.						

*reference category.

Lower general psychopathology PANSS score was significantly associated with higher spirituality (Beta = -0.61, *P* < 0.001). Increased general psychopathology PANSS score was significantly associated with higher fear of COVID-19 (Beta = 0.66, *P* = 0.010) (Table 7). The model to which the interaction between fear of COVID-19 and spirituality was added explained the variation in the general psychopathology PANSS better (R^2^ = 52%) than model 1 (R^2^ = 47%); the interaction between spirituality and fear of COVID-19 was significantly associated with lower general psychopathology PANSS score (Beta = -0.09, *P* < 0.001).

**Table 7:** Linear regression analysis with the general psychopathology Positive and Negative Syndrome Scale score as the dependent variable

		Unstandardized Beta	Standardized Beta	p	Confidence interval	
					lower bound	upper bound
Model 1	Fear of COVID-19 scale	0.66	0.23	**0.005**	0.20	1.11
	Age	0.04	0.03	0.708	-0.15	0.23
	Gender (male* vs female)	-10.22	-0.27	**0.003**	-16.80	-3.63
	Place of living	-4.66	-0.09	0.256	-12.75	3.43
	Marital status (married vs single*)	9.64	0.14	0.057	-0.28	19.57
	Education (complementary vs primary*)	2.06	0.05	0.580	-5.31	9.44
	Education (secondary vs primary*)	-7.57	-0.17	0.050	-15.15	0.01
	Education (university vs primary*)	-4.76	-0.10	0.263	-13.14	3.63
	Spirituality index scale	-0.61	-0.29	**0.001**	-0.96	-0.27
Adjusted R^2^ = 0.47						
Model 2	Fear of COVID-19 scale	3.91	1.38	**<0.001**	1.89	5.93
	Age	0.04	0.03	0.702	-0.15	0.22
	Gender (male* vs female)	-8.99	-0.24	**0.006**	-15.30	-2.67
	Place of living	-5.37	-0.11	0.170	-13.09	2.35
	Marital status (married vs single*)	6.33	0.09	0.197	-3.34	16.00
	Education (complementary vs primary*)	1.42	0.03	0.690	-5.62	8.45
	Education (secondary vs primary*)	-6.67	-0.15	0.071	-13.91	0.57
	Education (university vs primary*)	-3.87	-0.08	0.340	-11.87	4.14
	Spirituality index scale	0.85	0.41	0.077	-0.10	1.80
	Interaction Spirituality by Fear of Covid-19 scale	-0.09	-1.22	**0.001**	-0.14	-0.03
Adjusted R^2^ = 0.52						
Adjusted variables: Age, gender, place of living, education level and marital status.						

*reference category.

## DISCUSSION

This study highlighted that spiritual beliefs in patients with schizophrenia directly affected representations of illness and psychopathology. Spirituality was significantly inversely correlated with depression, anxiety, and psychosis. In patients with high fear of COVID-19, having low spirituality was significantly associated with higher depression, anxiety, and psychosis.

In line with recent studies (26,37), our findings revealed a positive correlation between anxiety and fear of COVID-19. This can be explained in many ways: first, due to the pandemic nature of COVID-19, fears were intensified worldwide and specifically among patients with schizophrenia (26). Second, many difficulties prevent patients with schizophrenia from following precautions, which has been associated with higher anxiety (37). In agreement with previous studies (24,25), the interaction between fear of COVID-19 and spirituality was correlated with lower anxiety. This is not surprising since the positive effect of spiritual beliefs on anxiety among individuals with schizophrenia and mental diseases is widely known (17,18). In stressful situations, patients use spiritual coping to facilitate problem-solving (19) and alleviate negative thoughts.

The inverse correlation of spirituality and depression observed in this study agrees with the results of previous studies (17,18). In addition, in patients with high fear of COVID-19, having low spirituality was significantly associated with higher depression, which corroborates the findings of recent studies (24,25). As explained above, the pandemic nature of the coronavirus has intensified fear of COVID-19 and increased the occurrence of depressive symptoms in patients with a psychiatric condition (8-10). These effects can be ameliorated by spiritual beliefs or behaviors (15); also, spirituality has been positively correlated with the psychological well-being of patients (15,38).

In addition, fear of COVID-19 positively correlated with increased total and general psychopathology PANSS scores. This was expected since previous studies confirmed an association between SARS-CoV-2 and psychosis (11,39). Many pathogenic pathways have been suggested to explain the correlation between COVID-19 and various psychotic symptoms. Regarding fear of COVID-19, restrictions associated with the pandemic, including hospital isolation protocols, may have caused a severe sensory deprivation, which possibly led to psychosis among COVID-19 patients (40).

In our study, spirituality was inversely correlated with total PANSS scores, which agrees with previous findings (41). In agreement with previous findings (23), we also found a significant association between low spirituality and higher psychosis in patients with high fear of COVID-19. During the COVID-19 pandemic, patients' spiritual beliefs positively affected their mental health (23). Patients with high spirituality had improved self-esteem and self-control, and were involved in activities, such as singing, meditating, and reading, that reduce the negative effect of the pandemic and reinforce positive symptoms (23).

There is a need to further evaluate the link between spiritual beliefs and symptoms of schizophrenia. A proper definition of spirituality in nursing mental health care practice and training will help nurses respond to patients’ mental and spiritual needs. This can help in the treatment of psychotic symptoms and pathological distortions related to spirituality. Specifically, during stressful conditions such as the COVID-19 pandemic and the economic and political crisis in Lebanon, clinicians should be more aware of the importance of spirituality in patient care procedures.

This study is subject to several limitations. First, the cross-sectional design prevents us from identifying causal relationships. Furthermore, the COVID-19 pandemic and related restrictions limited us from collecting data from all patients with schizophrenia admitted to the hospital; the sample size was relatively small although enough statistical power was reached for the bivariate and multivariable analyses. The Arabic versions of FACIT-SP12 and FCV-19 have not been previously validated in Lebanon. In addition, patients were chosen from a single hospital, which predisposes the study to selection bias. As patients may have overestimated or underestimated some questions, an information bias might also have been present. Because not all factors correlated with depression, anxiety, and psychosis were included in the questionnaire, such as treatment duration or treatment type, a residual confounding bias is expected. However, our study was the first to assess fear of COVID-19 and its association with psychopathology among patients with schizophrenia using different validated scales (LAS-10, MADRS, and PANSS). It was also the first to evaluate the interaction between fear of COVID-19 and spirituality among inpatients with schizophrenia.

Research is warranted to evaluate the effect of including spiritual therapy in the treatment of patients with schizophrenia. A challenge remains for clinicians to find a strategy how to encourage patients' spiritual behavior as part of patients' treatment.
